# Characteristics of Cancer in Subjects Carrying Lynch Syndrome-Associated Gene Variants in Taiwanese Population: A Hospital-Based Study in Taiwan

**DOI:** 10.3390/cancers16213682

**Published:** 2024-10-31

**Authors:** Yi-Peng Chen, Tzu-Hung Hsiao, Wan-Tzu Lin, Yi-Jun Liao, Szu-Chia Liao, Hsin-Ju Tsai, Yen-Ju Chen, Pei-Pei Jhan, Pei-Ying Kao, Ying-Cheng Lin, Han-Ni Chuang

**Affiliations:** 1 Division of Gastroenterology, Department of Internal Medicine, Taichung Veterans General Hospital, Taichung 40705, Taiwan; great800700@vghtc.gov.tw (Y.-P.C.); woantyy0025@vghtc.gov.tw (W.-T.L.); yjliao@vghtc.gov.tw (Y.-J.L.); scarkett@vghtc.gov.tw (S.-C.L.); tsaihj@vghtc.gov.tw (H.-J.T.); 2 Department of Medical Research, Taichung Veterans General Hospital, Taichung 40705, Taiwan; thsiao@vghtc.gov.tw (T.-H.H.); aoaichen@vghtc.gov.tw (Y.-J.C.); cuepeipei@vghtc.gov.tw (P.-P.J.); peggy9456@vghtc.gov.tw (P.-Y.K.); 3 Department of Public Health, Fu Jen Catholic University, New Taipei City 24205, Taiwan; 4 Institute of Genomics and Bioinformatics, National Chung Hsing University, Taichung 40227, Taiwan; 5 Department of Post-Baccalaureate Medicine, College of Medicine, National Chung Hsing University, Taichung 40227, Taiwan; 6 Division of Allergy, Immunology and Rheumatology, Department of Internal Medicine, Taichung Veterans General Hospital, Taichung 40705, Taiwan; 7 School of Medicine, National Yang Ming Chiao Tung University, Taipei 112304, Taiwan

**Keywords:** protein-truncating variant, germline mutation, Lynch syndrome, prostate cancer

## Abstract

Lynch syndrome (LS) is an autosomal dominant disorder characterized by increased risks of colorectal and endometrial cancers. LS is defined by pathogenic variants in mismatch repair (MMR) genes, including MLH1, MSH2, and MSH6. Data on the prevalence and associated cancer risks of LS in the Han Chinese population remain limited. In this study, using a broad biobank approach through the Taiwan Precision Medicine Initiative (TPMI), we identified LS-associated MMR gene variants within a cohort of 42,828 participants from a Taiwanese medical center. A total of 89 individuals were found to carry pathogenic MMR variants: MLH1 (n = 22, 25%), MSH2 (n = 47, 53%), and MSH6 (n = 20, 22%). The overall prevalence of MMR variants was calculated, and cancer incidence rates among carriers were determined. The prevalence of MMR variants in the study population was 1 in 481. The distribution of MLH1, MSH2, and MSH6 variants were 24.7%, 52.8%, and 22.5%, respectively. Cumulative cancer incidence rates of carriers were 40.9% for MLH1 carriers, 29.8% for MSH2, and 40% for MSH6. Among the 19 individuals who underwent colonoscopy screening, the prevalence of polyps was similar to that of the control group (adenoma detection rate: 32% vs 26%, *p* = 0.585). A meticulous analysis of the detected polyps in seven participants, considering factors such as location, size, morphology, and pathological features, showed no significant differences from controls. A significant cancer risk is associated with LS-related MMR variants in the Taiwanese population. The apparent under diagnosis of LS highlights the urgent need for enhanced surveillance and genetic counseling in this demographic. Our findings suggest that adjustments in the current screening protocols may be warranted to better identify and manage at-risk individuals.

## Simple Summary

Lynch syndrome (LS) is an autosomal dominant disorder linked to increased risks of colorectal and endometrial cancers, caused by pathogenic variants in MMR genes (MLH1, MSH2, MSH6). In a cohort of 42,828 participants from the Taiwan Precision Medicine Initiative (TPMI), 89 individuals carried MMR gene variants: 25% MLH1, 53% MSH2, and 22% MSH6, with a prevalence of 1 in 481. Cancer incidence rates were 40.9% for MLH1, 29.8% for MSH2, and 40% for MSH6 carriers. Colonoscopy screening revealed no significant differences in polyp prevalence compared to controls. The study underscores the need for improved LS diagnosis and surveillance in the Taiwanese population.

## 1. Introduction

Lynch syndrome (LS) is an autosomal dominant syndrome associated with greater risks for cancer, in particular colorectal cancer (CRC) and gynecological cancers. LS is caused by mutations in mismatch repair genes (MMR), including MSH2, MLH1, MSH6, PMS2 and deletions in the EPCAM gene [1,2]. LS represents 2–4% of all colorectal cancers (CRCs) and is the most common hereditary form of CRC [3,4].

Most LS patients are typically identified through a genetic test after meeting certain clinical diagnostic criteria, such as the Amsterdam II criteria or according to the revised Bethesda guidelines [5,6]. These criteria are based primarily on personal and family cancer history, age of onset, and molecular features of tumors, but they have low sensitivity ranging from 25% to 50% [7,8]. As a result, LS is likely underdiagnosed, leading to missed identification of some high-risk individuals [9]. These individuals include those with ambiguous or incomplete family medical histories, as well as those whose initial presentation does not involve the more commonly associated cancers such as colorectal and endometrial cancers.

Many studies indicated that LS may be more common than previously thought, with prevalence estimated between 1 in 180 and 1 in 279 [10,11,12]. Therefore, it is important to acquire knowledge on the prevalence, phenotypes, and cancer risks in individuals with MMR genetic variants in the general population.

For LS patients, routine colonoscopy performed annually or biannually together with polypectomy markedly reduces CRS risk [13,14,15]. Jarvinen et al. showed that in LS patients, their CRC risks can be reduced by half with regular colonoscopy [16]. The above findings indicated that at least some CRCs in these patients arise from precursor lesions that can be detected by colonoscopy and removed. On the other hand, more recent studies on LS patients reported no higher prevalence of premalignant lesions, such as advanced adenoma and serrated sessile lesions was not increased in LS patients [17]. It remains unclear whether individuals with MMR variants, who have not been previously diagnosed with LS, have a higher prevalence of premalignant colon lesions.

In this study, we evaluated the prevalence of cancers associated with LS among carriers of mismatch repair (MMR) gene variants in the Han-Chinese, a major ethnicity of the Chinese population. We further assessed the age at onset of LS-related cancers across different MMR gene variants. We also prospectively recruited carriers of MMR variants who had not been previously diagnosed with LS-related cancers and subjected them to endoscopic surveillance to detect colorectal neoplasms.

## 2. Materials and Methods

### 2.1. Enrollment of Participants and Identification of Lynch Syndrome Associated Variants

**Figure 1:**
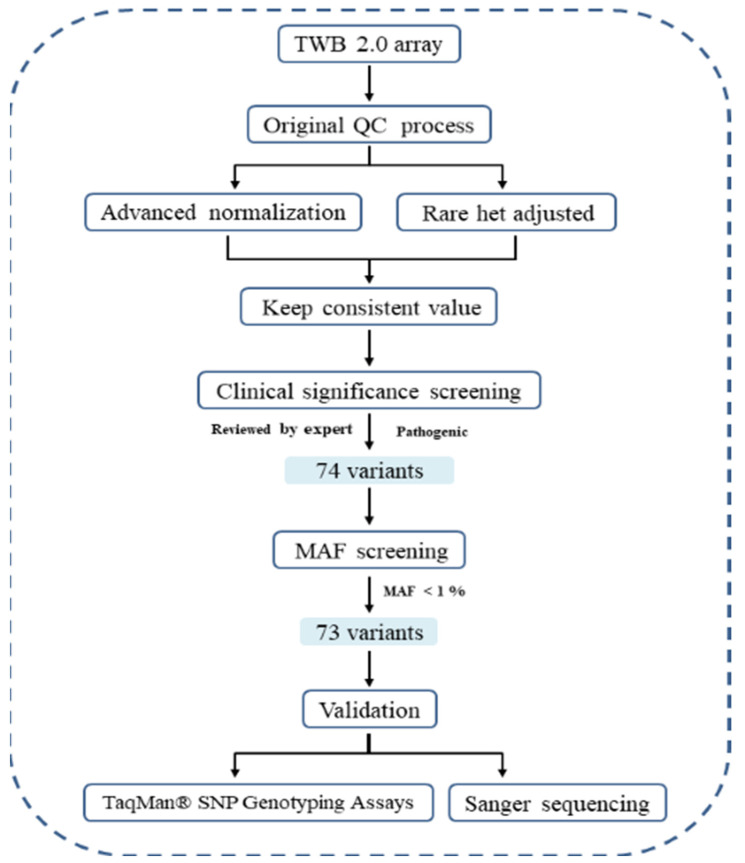
Genotype calling, candidate variants screening, and validation. QC—Quality control; MAF—minor allele frequency; SNP—single nucleotide polymorphism.

Between June 2018 and December 2021, we enrolled 42,828 individuals aged 20 years and older at a medical center in Taiwan, in collaboration with the Taiwan Precision Medicine Initiative (TPMI) overseen by the Academia Sinica, Taiwan. We excluded individuals who had been previously diagnosed with Lynch Syndrome-related cancers and had undergone a colonoscopy within the past two years. We gathered their detailed information from medical records and blood tests. All participants received genotyping based on an Affymetrix TWB 2.0 SNP chip, which includes variants associated with LS. These variants have been identified as pathogenic and underwent expert review in the ClinVar database [18]. Initially, we identified 74 pathogenic LS-associated variants, but removed one variants with an MAF (Minor allele frequency) <1%. A total of 73 LS-associated variants, which were subsequently validated through SNP genotyping or Sanger sequencing. Ultimately, 22 validated variants were retained for further analysis (Figure 1). These variants ware prevalent among the Taiwanese population, as indicated by data from the Taiwan biobank (TaiwanView: https://taiwanview.twbiobank.org.tw/, accessed on 28 October 2024). Our study adhered to the ethical principles outlined in the Declaration of Helsinki, with protocol approved by the Institutional Review Board of the Taichung Veterans General Hospital (ethical approval code: CF23286A). All participants who were prospectively recruited provided signed informed consent. However, for the matched control group, the Institutional Review Board of the Taichung Veterans General Hospital waived the requirement for informed consent, as the data collection was retrospective. Anonymous medical record data were acquired from the Clinical Informatics Research and Development Center of Taichung Veterans General Hospital after eliminating any identifying information.

### 2.2. Genotyping and Quality Controls

Genetic data of participants were derived from single nucleotide polymorphism (SNP) arrays acquired through the Taiwan Precision Medicine Initiative (TPMI). A total of 714,461 probe-sets were tailored on the Axiom Genome-Wide TWB 2.0 Array Plate by Affymetrix, Santa Clara, CA, USA. Typically, most rare diseases are associated with rare pathogenic variants, which manifest with a minor allele frequency (MAF) of <1% in the population. To address the challenge of low accuracy and high false positive rates in detecting rare variants using SNP arrays [19], we implemented a specific calling approach based on our previously established method [20]. Genotype calling relied on Affymetrix^®^ Power Tools (APT), a command-line software from Santa Clara, CA, USA. We applied advanced techniques of normalization and adjustment algorithms to rare heterozygotes. Rare variants were only designated provided that they were identified by both methods. Otherwise, they were classified as wild type.

### 2.3. Prospective Screening Colonoscopy Among Variant-Positive Participants

We recruited individuals without LS-associated cancers and conducted a screening colonoscopy for each of them. Among the contacted participants, 13 individuals declined the recruitment because they had had a colonoscopic examination within the past few years. In addition, 20 participants were unwilling to receive a colonoscopy. Eventually, we successfully recruited 19 participants, of whom 17 had never received a colonoscopic examination, while the remaining 2 individuals had their last colonoscopy more than 5 years ago.

### 2.4. Detection of Colon Polyps in Variant-Positive Case-Control Studies

The screening colonoscopic examinations were performed by two experienced endoscopists, each with the experience of 3000 colonoscopy procedures. The right side of the colon was routinely examined twice. The net withdrawal time was >6 min for all participants. The Boston Bowel Preparation Scale (BBPS) score was used to rate the quality of preparation [21]. All detected polyps during the procedure were removed. The definition of the proximal colon lesions was a location proximal to the sigmoid colon. The flat morphology was nonpolypoid type (0-IIa, IIb, IIc) according to the Paris classification [22]. Lesions were later examined histologically by two gastrointestinal (GI) pathologists. The definition of advanced adenoma was an adenoma ≥10 mm in diameter, or with villous appearance in histology, or high-grade dysplasia. Serrated polyps were categorized into hyperplastic polyps (HPs), sessile serrated lesion (SSLs) either with or without dysplasia, and traditional serrated adenoma (TSAs), according to the WHO classification of tumors (2019) [23]. The rates of different polyp types were compared to 1:10 age–gender–BMI matched individuals without a history of CRC, based on the results of their colonoscopy screening at our health promotion center.

### 2.5. Statistical Analyses

Descriptive statistics were employed to describe participants and polyp characteristics. The Mann–Whitney U test was used to assess the differences for continuous variables between participants with pathogenic variants and matched controls. The Chi-square test and Fisher’s exact test were used to determine associations between categorical variables. Analyses were performed using the Statistical Package for the Social Sciences (IBM SPSS version 22.0; International Business Machines Corp, New York, NY, USA).

## 3. Results

### 3.1. MMR Mutations Identified Among the TPMI Participants

The group of participants drawn from the TPMI cohort provided valuable insights into the presence of pathogenic variants within *MMR* genes. Based on the clinical significance reports and data from the ClinVar database, we selected 73 variants in *MMR* genes using the TWB2.0 chip. These variants were validated through the TaqMan SNP genotyping assay and Sanger sequencing. Ultimately, we identified 20 confirmed pathogenic variants, including 8 in *MLH1*, 7 in *MSH2*, and 5 in *MSH6* (Table 1).

In 42,828 TPMI participants, we found 89 individuals carrying the pathogenic variants. MLH1 was found in 22 individuals (24.7%), MSH2 in 47 individuals (52.8% each), and MSH6 in 20 individuals (22.5%). The overall prevalence of TPMI participants harboring MMR variants was 1 in 482 individuals (89 subjects within 42,828 samples), emphasizing the significance of genetic screening and further investigation within this specific population (Table 2).

### 3.2. Demographics and Clinical Characteristics of Participants Harboring Pathogenic MMR Variants and VUS

Here, we present a detailed analysis of those subjects in the TPMI cohort identified to have pathogenic MMR variants. As shown in Table 2, a total of 89 individuals were identified with pathogenic MMR variants. Their median age was 60.4 years, with a range from 23 to 89 years, and the group consisted of 43 males and 46 females. Of these participants, 31 (34.8%) had a personal history of cancer, including17 males and 14 females. The average age at cancer diagnosis was 61.5 years (range: 26 to 89 years). Additionally, nine participants reported a family history of cancer.

In our cohort, the patients carrying the MLH1 variants were 64% male and 36% female, while those with MSH2 and MSH6 variants had equal gender distribution. Their average age of cancer diagnosis for carriers of MLH1, MSH2, and MSH6 was 57.3, 64.1, and 59.7 years, respectively (Table 2). A total of 34.8% (n = 31) of participants had a personal history of cancer, including 40.9% (n = 9) of MLH1 carriers, 29.8% (n = 14) of MSH2 carriers, and 40% (n = 8) of MSH6 carriers. The average age at cancer diagnosis for the entire group was 61.5 years (Table 2). 

In addition, MMR carriers of our cohort, colorectal cancer was the most common type, constituting 18.0% (n = 16) of the total cohort, with the highest prevalence in MLH1 carriers (36%, n = 8). Endometrial cancer is less common, observed in 2.2% (n = 2) of participants.

A family history of cancer was reported by 22.5% (n = 20) of the participants, with colorectal cancer being the most frequent, accounting for 7.9% (n = 7). The group of MLH1 carriers had the highest proportion of participants with a family history of cancer at 23% (n = 5), followed by the group of MSH6 carriers at 20% (n = 4), while the MSH2 group had the lowest at 13% (n = 6) (Table 2).

Among these 89 participants, 24.7% had a personal history of Lynch Syndrome (LS)-related cancers, and 7.9% reported a family history of such cancers (Table 2). Of the 14 female participants, 2 (14.3%) had a personal history of endometrial cancer, although no family history of endometrial cancer was reported. Further analysis revealed variations in cancer rates among the different MMR variants. Participants with pathogenic MLH1 variants had the highest rate of cancer at 40.9%, followed closely by those with pathogenic MSH6 variants at 40%, and those with pathogenic MSH2 variants at 28% (Table 2, “Personal cancer history”). Specific cancer types also varied by MMR variant. For example, 18.2% (2 out of 11) of females with pathogenic MSH6 variants had endometrial cancer, while 36% of those with pathogenic MLH1 variants had a personal history of CRC, and 23% had a family history of CRC. These findings highlight the diverse clinical manifestations associated with different MMR variants.

### 3.3. MMR Variant Carriers Have Higher Cancer Rates in Individuals over 60 Years of Age

**Figure 2:**
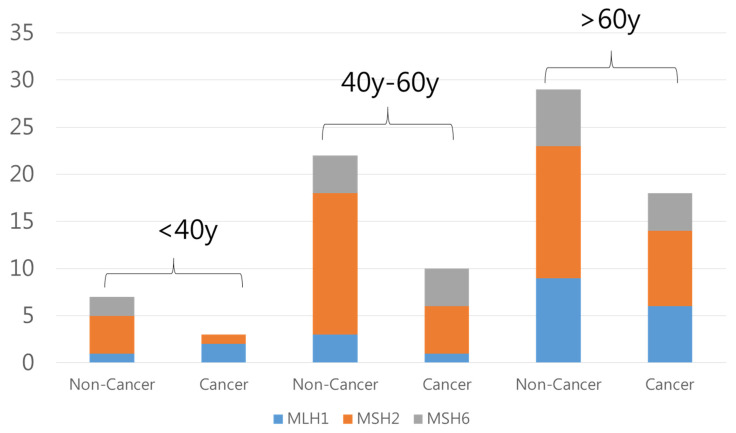
Distribution of MLH1, MSH2, and MSH6 gene variants by age group and cancer status. The distribution of MLH1, MSH2, and MSH6 gene variants among participants in our cohort, stratified by age group (<40 years, 40–60 years, and >60 years) and cancer status (non-cancer and cancer). The vertical bars represent the number of individuals carrying pathogenic variants in each gene, with the color-coded segments indicating the specific gene affected: MLH1 (blue), MSH2 (orange), and MSH6 (gray).

We stratified the distribution of MLH1, MSH2, and MSH6 gene variants in our cohort by age groups (>40 years, 40 to 60 years, and <60 years) and cancer status. In the >40 years group, a higher proportion of non-cancer individuals was observed (n = 7), particularly among those with the MSH2 variant (n = 4), resulting in a cancer to non-cancer ratio of 3:7 (0.43). In the 40 to 60 years group, non-cancer individuals were predominant, especially among those with the MSH2 variant (n = 15), with a cancer to non-cancer ratio of 10:22 (0.45). The <60 years group showed a similar trend, particularly among those with the MSH2 variant (n = 14), yielding a cancer to non-cancer ratio of 18:29 (0.62). These results suggest that the cancer ratio among carriers of MMR variants increases with age (Figure 2). 

### 3.4. Only Two Individuals Were Clinically Diagnosed with Lynch Syndrome in MMR Variant Carriers

Despite a notable prevalence of LS-related cancers within the 89 individuals carrying MMR variants, only 2 of them had a formal diagnosis of LS. In one case, a male participant with the rs63750909 presented with advanced sigmoid cancer. Immunohistochemistry analysis of the MMR proteins revealed the absence of MSH6. The diagnosis of LS was subsequently confirmed based on this finding, along with a family history of colorectal cancer at a young age. In a parallel case, the second individual diagnosed with LS carried the SNP rs587778882. This participant reported a family history of LS, and a personal history of both CRC and gastric cancer. More importantly, immunohistochemistry screening was not conducted at the time of the diagnosis in 2014. These two cases underscore the challenges in identifying LS, even in the presence of LS-related cancers, emphasizing the need for comprehensive genetic and clinical assessments in the context of MMR variants.

### 3.5. Prevalence of Different Colon Neoplasms in Individuals with MMR Mutations

Screening colonoscopy was conducted on 19 subjects. As shown in Table 3, their mean age was 55.4 ± 13.3 years old. During colonoscopy, the recruited participants exhibited a median Boston Bowel Preparation Scale (BBPS) score of 9, along with a >10 mind withdrawal time. Within this cohort, we identified one sessile serrated lesion (SSL) without dysplasia (5%) and 6 hyperplastic polyps (32%). Also, six patients presented with adenomas or equivalent to a 32% detection rate.

A comparison of the results from MMR mutation carriers (n = 19) with age–gender–body mass index (BMI)-matched controls (n = 190) from the health promotion center highlighted similar outcomes despite the recruited MMR mutation carriers demonstrating superior BBPS scores and longer withdrawal times. A meticulous analysis of the detected polyps shows the results in Table 4, covering various factors such as location, size, morphology, and pathological features. Noteworthy observations included the location of adenomas being in the distal part of the colon (57%) with an average size of 5.3 ± 3.3 mm. Flat morphology was observed in 57% of cases, and only one adenoma (14%) met the criteria for an advanced adenoma (size ≥ 10 mm). One SSL (5%) was detected in the MMR variant carriers group, compared with 3 SSLs (2%) in the control group. The mean size of SSLs in the MMR variant carriers group was 4.0 ± 0 mm, compared with 5.0 ± 1.0 mm in the control group (*p* = 0.478), with no significant statistical difference. We found 6 HPs or other non-neoplastic polyps in the MMR variant carriers group (32%), compared to 95 (50%) in the control group. Also, 33% of HPs or other non-neoplastic polyps in the MMR variant carriers group (n = 2) were located in the proximal colon, compared with 32% (n = 30) in the control group (*p* = 1.000). The average size of HPs or other non-neoplastic polyps in the MMR variant carriers group was 2.8 ± 0.75 mm, compared with 3.62 ± 1.86 mm in the control group (*p* = 0.306), with no statistically significant difference. All HPs or other non-neoplastic polyps in the MMR variant carriers group exhibited flat or elevated morphology (n = 6), compared with 88% (n = 84) in the control group (*p* = 1.000) (Table 4). Comparative analysis with controls revealed no significant differences in the prevalence, location, size, morphology, or pathological features of various polyp types. 

## 4. Discussion

In this study, we have identified individuals with MMR variants from a large, ancestrally homogeneous biobank in Taiwan. Among this group, we observed that 18% of individuals had colorectal cancer, and 24.7% had LS-related cancers. Those carrying pathogenic MLH1 or MSH6 variants had a cancer risk exceeding 40%. In the later part of the study, we conducted colonoscopies on participants with these variants, but they had no LS-related cancers diagnosis. Among the 19 participants, we found a comparable prevalence of various types of colon polyps when compared with the control group.

LS is generally considered a disease with prevalence being underestimated [24]. Epidemiology studies are primarily focused on Western populations [25], with few dedicated to Eastern population. In our study, the overall prevalence of pathogenic MMR variants was found to be 1 in 481 individuals. Among individual MMR variants, MSH2 exhibited the highest prevalence (1 in 912), while MSH6 had the lowest (1 in 2144). These findings are in line with prior studies on LS involving individuals with hereditary cancers and those conducted using diverse genomic biobanks [24]. The estimated overall prevalence of LS ranges from 1 in 300 to 1 in 440, depending on ethnicities [26], similarity to our results. Pathogenic mutations in MLH1, MSH2, and MSH6 are the three most common variants, with frequencies ranging from 1 in 750 to 1 in 2800 [11,26]. Our results on the overall prevalence of LS in Taiwan, as determined through population-based genetic screening, align with these established estimates.

In this study, we utilized an unselected biobank, predominantly of non-diagnosed individuals with hereditary diseases. Despite this, the prevalence of colorectal cancers and other malignancies associated with LS was comparable to previous findings from other large prospective datasets that specifically recruited participants previously diagnosed with LS [26]. For instance, in our current study, 3% of participants with a pathogenic MLH1 variant were found to have colorectal cancer, a percentage consistent with a previous report from the prospective LS database, indicating a colorectal cancer risk in MLH1 carriers ranging from 32.2% to 45.2% by the age of 60 [27]. Similar outcomes were observed in participants with MSH6 variants. Despite MSH6 once being considered a variant with lower penetrance compared with MLH1 and MSH2 [24,28], our results revealed a significant risk of various LS-related cancers among MSH6 carriers. This finding is consistent with a prior report involving participants from diverse ancestries, emphasizing MSH6 having the highest cancer risk among various MMR mutations [9]. In our current study, 55% of women (11 in 20) with pathogenic MSH6 variants had a prior diagnosis of endometrial cancer. Previous studies have indicated that, in comparison to other MMR mutations, the cumulative risk of endometrial cancer was highest among MSH6 carriers [29,30,31].

Our study revealed a notable cancer risk among individuals with MMR mutations, with the majority of them being unaware of their risk status. Only two of the MMR mutation carrying individuals were diagnosed with LS. This finding is consistent with a prior report on a lack of documented LS diagnoses in the most MMR carriers [10]. Increasing awareness of such underdiagnosis is crucial, and various approaches have been suggested to improve this situation [32]. For instance, current guidelines advocate for universal screening in patients either colorectal cancer (CRC) or endometrial cancer (EC), using immunohistochemistry and/or m patients with microsatellite instability tests to detect loss of MMR function [8,25,33]. However, these tests may still miss some LS patients, especially those without CRC or EC. Based our current results and previous reports from multigene panel screenings in the general population, we have identified a significant cancer risk in individuals carrying MMR variants. Even in the absence of a formal diagnosis of hereditary cancer syndrome, considering the implementation of aggressive surveillance programs for these MMR mutation carriers may prove clinically worthwhile. However, the prevalence of colon neoplasms, specifically sessile serrated lesions (SSL), in patients with LS remains undetermined. 

In the second part of our current study, we conducted screening colonoscopy on participants with MMR mutations who lacked a prior diagnosis of LS-related cancers. Of the 19 patients recruited, 17 never received colonoscopy before. The procedures were carried out by two experienced endoscopists. Regular screening colonoscopy with polypectomy has significant reductions in CRC risk [16]. Nonetheless, the occurrence of colon neoplasms, such as SSL, in LS patients remains a contentious issue [17]. Our colonoscopic examinations on MMR mutation carriers without prior LS-related cancer diagnoses revealed similar rates of polyp detection compared with matched controls. This outcome aligns with findings from other prospective studies conducted on LS patients [17]. Jasper et al. reported comparable detection rates of adenoma, hyperplastic polyps, SSL, and TSAs between LS patients and the control group. It is worth noting that the median BPPS score was excellent, the median withdrawal time exceeded 10 min, and the right side of the colon was routinely examined twice, utilizing additional image-enhanced endoscopy, we achieved with an adenoma detection rate of 37%. These results indicate satisfactory colonoscopy quality, meeting high standards compared with other studies [34]. 

This is the first LS study conducted in Southeast Asia using a multigene panel approach. By genetic screenings on a large population, we identified a significant number of clinically underdiagnosed patients [23,24]. Our study further assessed individual cancer risk with variants of uncertain significance, revealing an elevated risk of LS-associated cancer in these individuals. As evidence accumulates, some variants of uncertain significance may become pathogenic. Lastly, we included individuals with MMR variants and performed prospective colonoscopy on those without previous regular screenings, ensuring that the polyp detection rate was not influenced by prior polypectomy.

Limitations of our study are as follows. Firstly, the recruitment from TPMI was mainly hospital-based, with potential sampling bias. Despite this, our findings aligned with other population-based data, and the cancer risk in our study participants overlapped with that reported in other populations. Secondly, the exclusion of all PMS2 alleles during the initial screening process had limited our ability to assess clinical impacts associated with this specific gene. Finally, while our high-quality colonoscopy in MMR mutation carriers showed a comparable prevalence of various polyps, the natural course of colorectal cancer development in LS remains unclear. Further longitudinal follow-up studies are needed in this population.

In conclusion, our study demonstrated that a genomics-first approach can effectively identify individuals with an elevated risk of cancer in Taiwan. A noteworthy finding is that a significant number of these individuals had not been formally diagnosed with LS, even when presenting with LS-associated cancers. Furthermore, colonoscopic surveillance in MMR mutation carriers detected polyps at rates similar to those observed in controls. This highlights the potential effectiveness of genetic screening in identifying individuals at risk, emphasizing the importance of proactive surveillance for early detection and intervention in those with MMR mutations.

## 5. Conclusions

The substantial cancer risks associated with Lynch syndrome-related MMR gene variants (MLH1, MSH2, MSH6) in the Taiwanese population. Despite the high prevalence of these variants, under diagnosis of Lynch syndrome remains a critical issue. The lack of significant differences in polyp detection between carriers and controls suggests that current screening protocols may not fully capture the cancer risk in this population. These findings emphasize the need for enhanced genetic counseling, more comprehensive surveillance strategies, and potentially revised screening guidelines to better identify and manage at-risk individuals.

## Figures and Tables

**Table 1:** Pathogenic variants in MMR genes identified among TPMI participants.

Position	rsID	Gene ^a^	Variant Type	HGVSc	HGVSp	Number of Participants
chr3:36993610-36993614	rs63750822	MLH1	Indel	c.63_67del	p.Ala21_Glu23fs	1
chr3:36993651-36993652	rs587778882	MLH1	Indel	c.104_105 insAA	p.Met35_Ile36fs	2
chr3:37004444	rs63750781	MLH1	SNV	c.350 C>A, G,T	p.Thr117 Lys, Arg, Met	1
chr3:37012099-37012100	rs587779032	MLH1	MNV	c.68-6_70del	p.His23del	1
chr3:37017500-37017510	rs587779041	MLH1	Indel	c.17_17+1del	p.Arg6Gln	3
chr3:37028926	rs587778925	MLH1	Indel	c.1552del	p.His518fs	5
chr3:37047632-37047641	rs63751247	MLH1	Indel	c.1845_1854 del	p.Leu615_Lys618del	2
chr3:37048973	rs63751275	MLH1	SNV	c.2059 C>A, G, T	p.Arg687, Gly, Trp	7
chr2:47403273	rs63751246	MSH2	SNV	c.82G>A, G	p.Glu28Lys, Ter	14
chr2:47412459	rs587779174	MSH2	Indel	c.691del	p.Asp231fs	1
chr2:47429783-47429784	rs63750516	MSH2	Indel	c.1119del	p.Arg373fs	2
chr2:47429794	rs63750267	MSH2	SNV	c.1129C>G, T	p.Gln377Glu, Ter	2
chr2:47466697-47466700	rs63749930	MSH2	Indel	c.1552_1553del	p.Gln518fs	1
chr2:47471020	rs267607974	MSH2	Indel	c.1717del	p.Ala573fs	26
chr2:47475053-47475054	rs63750495	MSH2	Indel	c.1788_1789del	p.Asn596fs	1
chr2:47799172-47799174	rs63750439	MSH6	Indel	c.1190_1191del	p.Tyr397fs	2
chr2:47799427	rs63750909	MSH6	SNV	c.1444C>A, G, T	p.Arg482, Gly, Ter	3
chr2:47799617	rs63749874	MSH6	Indel	c.1634_1637del	p.Lys545fs	12
chr2:47800130-47800136	rs267608058	MSH6	Indel	c.2150_2153del	p.Val717fs	2
chr2:47800996	rs63750563	MSH6	SNV	c.3013C>A, G, T	p.Arg1005, Gly, Ter	1

^a^ cDNA position provided for MLH1 ENST00000231790 (NM_000249.3), MSH2 ENST00000233146 (NM_000251.2), MSH6 ENST00000234420 (NM_000179.2). Abbreviations: rsID—reference SNP ID; HGVS—Human Genome Variation Society; SNV—single nucleotide variant; MNV—multiple nucleotide variant; Indel—insertion/deletion; MMR—mismatch repair.

**Table 2:** Clinical Characteristics of 89 TPMI Participants with Pathogenic variant in MLH1, MSH2, MSH6.

	Pathogenic MMR	MLH1	MSH2	MSH6
TPMI Participants	89	22 (24.7%)	47 (52.8%)	20 (22.5%)
Median (range) age, years	60.4 (23~89)	62.6 (24~78)	59.1 (23~89)	60.5 (23~82)
Sex				
Male	43 (48%)	14 (64%)	20 (43%)	9 (45%)
Female	46 (52%)	8 (36%)	27 (57%)	11 (55%)
Personal Cancer	30 (33.7%)	9 (41%)	13 (28%)	8 (40%)
Mean Age at diagnosis of cancer (range )	61.5 (26~89)	57.3 (26~71)	64.1 (32~89)	59.7 (43~78)
Male	17 (54.8%)	7 (22.6%)	7 (22.6%)	3 (9.7%)
Female	14 (45.2%)	2 (6.5%)	7 (22.6%)	5 (16.1%)
Personal Cancer Type (n = 31)				
Colorectal cancer	16 (18.0%)	8 (36%)	6 (13%)	2 (10%)
Endometrial cancer	2 (2.2%)	0	0	2 (10%)
Any LS-related cancer	22 (24.7%)	9 (41%)	7 (15%)	6 (30%)
Family Cancer history	20 (22.5%)			
Colorectal cancer	7 (7.9%)	5 (23%)	2 (4%)	0
Endometrial cancer	0	0	0	0
Any LS-related cancer	7 (7.9%)	5 (23%)	2 (4%)	0
Any cancer	15 (16.9%)	5 (23%)	6 (13%)	4 (20%)

Abbreviations: LS—Lynch syndrome; MMR—mismatch repair. LS-related cancers include tumors of the colorectum, endometrium, stomach, small bowel, ovaries, pancreas, ureter, renal pelvis, biliary tract, brain, and sebaceous gland as well as keratoacanthomas.

**Table 3:** Baseline characteristics of Lynch syndrome patients and endoscopic detection rates of polyps.

	MMR Variant Carrier (n = 19)	Control (n = 190)	*p* Value
Age, y, mean ± SD	55.4 ± 13.3	54.5 ± 11.6	0.637
Female gender, n (%)	10 (53%)	98 (52%)	0.930
MMR mutation, n (%)			
MLH1	2 (11%)	0	
MSH2	13 (63%)	0	
MSH6	4 (21%)	0	
BBPS, Median (IQR)	9 (9-9)	8 (7–8)	<0.001 ***
Withdrawal time	10′14″	07′06″	<0.001 ***
Adenomas, n (%)			
≥1 Adenoma	6 (32%)	49(26%)	0.585
Serrated polyps, n (%)			
≥1 SSL	1 (5%)	3 (2%)	0.319
≥1 high-risk SSL	0	1 (0.5)	1.000
≥1 HP	6 (32%)	67 (35%)	0.748
≥1 TSA	0	0	

Abbreviations: MMR—mismatch repair; n—number; SD—standard deviation; BBPS—Boston Bowel Preparation Scale; IQR—interquartile range; SSL—sessile serrated lesion; HP—hyperplastic polyp.; TSA—traditional serrated adenoma. ***, *p* < 0.001.

**Table 4:** Comparative analysis of colonic polyp pathology in MMR variant carriers and control subjects.

	MMR Variant Carrier (n = 19)	Control (n = 190)	*p* Value
Adenomas	7	71	
Proximal location, n (%)	3 (43%)	46 (65%)	0.414
Size, mean ± SD	5.3 ± 3.3	4.66 ± 3.06	0.610
Flat (elevated) morphology, n (%)	4 (57%)	36 (51%)	1.000
Advanced adenoma, n (%)	0	0	
HGD	0	0	
Villosity, n (%)	0	4 (6%)	1.000
≥10 mm, n (%)	1 (14%)	4 (6%)	0.383
SSLs, n (%)	1 (5%)	3 (1.6%)	
Proximal location, n (%)	0	2 (1.1%)	1.000
Size in mm, mean ± SD	4.0 ± 0	5.0 ± 1.0	0.478
Flat (elevated) morphology, n	0	3 (1.6%)	0.250
High-risk SSL, n	0	1 (0.5%)	1.000
HPs and other non-neoplastic polyps	6 (31.6%)	95 (50%)	
Proximal location, n (%)	2 (10.5%)	30 (15.8%)	1.000
Size, mean ± SD	2.8 ± 0.75	3.62 ± 1.86	0.306
Flat (elevated) morphology	6 (31.6%)	84 (44%)	1.000

Values are polyp numbers (%), unless otherwise indicated. Abbreviations: MMR—mismatch repair; n—number; SD, standard deviation; HGD, High-grade dysplasia; SSL, sessile serrated lesion; HPs, hyperplastic polyps.

## Data Availability

Data are contained within the article.
